# Direct and indirect cost of attempted suicide in a general hospital: cost-of-illness study: Custo direto e indireto de tentativas de suicídio em um hospital geral: estudo de custo de doença

**DOI:** 10.1590/1516-3180.2014.8491808

**Published:** 2014-12-19

**Authors:** Sara Maria Teixeira Sgobin, Ana Luisa Marques Traballi, Neury José Botega, Otávio Rizi Coelho

**Affiliations:** I MSc. Collaborating Physician, Department of Medical Psychology and Psychiatry Universidade Estadual de Campinas (Unicamp), Campinas, São Paulo, Brazil.; II MD. Collaborating Physician, Department of Medical Psychology and Psychiatry Universidade Estadual de Campinas (Unicamp), Campinas, São Paulo, Brazil.; III PhD. Titular Professor, Department of Medical Psychology and Psychiatry, Universidade Estadual de Campinas (Unicamp), Campinas, São Paulo, Brazil.; IV PhD. Titular Professor, Department of Cardiology, Universidade Estadual de Campinas (Unicamp), Campinas, São Paulo, Brazil.

**Keywords:** Fees and charges, Suicide, attempted, Costs and cost analysis, Health care costs, Hospital costs, Honorários e preços, Tentativa de suicídio, Custos e análises de custo, Custos de cuidados de saúde, Custos hospitalares

## Abstract

**CONTEXT AND OBJECTIVE:**

Knowledge of socioeconomic impact of attempted suicide may sensitize managers regarding prevention strategies. There are no published data on this in Brazil. The aim here was to describe the direct and indirect costs of care of hospitalized cases of attempted suicide and compare these with the costs of acute coronary syndrome cases.

**DESIGN AND SETTING:**

Cost-of-illness study at a public university hospital in Brazil.

**METHOD:**

The costs of care of 17 patients hospitalized due to attempted suicide were compared with those of 17 acute coronary syndrome cases at the same hospital, over the same period. The direct costs were the summation of the hospital and out-of-hospital costs resulting from the event, determined from the medical records. The indirect costs were estimated through the human capital lost. The Mann-Whitney test and analysis of covariance (ANCOVA) with transformation adjusted for age were used for comparisons.

**RESULTS:**

The average costs per episode of attempted suicide were: direct cost, US$ 6168.65; indirect cost, US$ 688.08; and total cost, US$ 7163.75. Comparative analysis showed a difference between the indirect costs to family members, with significantly higher costs in the attempted suicide group (P = 0.0022).

**CONCLUSION:**

The cost of care relating to attempted suicide is high and the indirect cost to family members reinforces the idea that suicidal behavior not only affects the individual but also his social environment.

## RESUMO

**CONTEXTO E OBJETIVO:**

O conhecimento do impacto socioeconômico das tentativas de suicídio pode sensibilizar gestores para programas de prevenção. No Brasil, não há dados publicados a esse respeito. O objetivo foi descrever os custos direto e indireto dos cuidados com as tentativas de suicídio de pacientes internados no hospital de uma universidade pública, e compará-los aos custos de casos de síndrome coronariana aguda.

**TIPO DE ESTUDO E LOCAL:**

Estudo de custo de doença em hospital universitário público no Brasil.

**MÉTODO:**

Os custos dos cuidados de 17 pacientes hospitalizados por tentativas de suicídio foram comparados aos custos de 17 síndromes coronarianas agudas de pacientes internados no mesmo hospital, no mesmo período. Os custos diretos foram o somatório de custos hospitalares e extra-hospitalares decorrentes do evento, determinados por registro hospitalar. Os custos indiretos foram estimados através da perda de capital humano. Teste de Mann-Whitney e análise da covariância (ANCOVA) com transformação por idade foram utilizados para comparação.

**RESULTADOS:**

As médias de custos por episódio de tentativa de suicídio foram: custo direto individual, US$ 6168,65; custo indireto individual, US$ 688,08; e custo total, US$ 7163,75. A análise comparativa indica diferença entre os custos indiretos a familiares dos dois fenômenos, com custos significativamente mais elevados no grupo de tentativas de suicídio (P = 0,0022).

**CONCLUSÃO:**

O custo dos cuidados com tentativa de suicídio é elevado e o custo indireto a familiares reforça a ideia de que o comportamento suicida não afeta apenas o indivíduo, mas também seu ambiente social.

## INTRODUCTION

More than mortality and prevalence alone, the burden of disease has become an important indicator of a population’s health. It can be described as the impact of a health problem on the population. According to the World Health Organization, suicide deaths around the world represented 1.5% of the global burden of disease in 2002. For 2020, it has been estimated that there will be 1.53 million suicides, representing 2.4% of the overall burden of disease.^1^ In cases of mental distress associated with nonfatal attempted suicide and suicide ideation, disability measures have yet to be developed.^2^ Knowledge of the financial burden of suicide and attempted suicide may be a strong motivator for public administrators to implement prevention strategies.^3^^,^^4^^,^^5^

The direct and indirect costs are those most commonly examined in studies.^6^^,^^7^^,^^8^ Direct costs are those directly linked to the disease. They are the summation of all the costs generated through the individual’s illness, such as outpatient treatment, daily hospital care, medications, examinations, medical fees, rehabilitation, transportation for treatment and procedures.^9^^,^^10^^,^^11^ Indirect costs include the economic loss or the years of healthy life lost as a result of suicide. These include damage to the patient and/or family members/caregivers when they cease their professional functions for an indefinite or definitive period of time as a result of absences from work, early retirement or death.^10^^,^^11^

The suicide rate in Brazil is 5.1 cases per 100,000 inhabitants.^12^ Although this is not among the highest rates in the world,^13^ it does result in a large number of deaths due to suicide because of the country’s large population: in 2011, there were 9,852 suicides.^14^ In addition to its emotional and social impact,^15^^,^^16^^,^^17^ premature death due to suicide has a large economic impact. In Brazil, a study on the indirect cost of suicide estimated that the total loss due to suicide was R$ 1.3 billion (US$ 720,000) in 2001.^18^

A New Zealand study on 460 suicides and 5,095 attempted suicides that occurred in 2002 showed that the total direct costs (considering the summation of all events) of suicide were less than the direct costs of attempted suicide (US$ 4,694,000 and US$ 19,029,000, respectively). However, over the long term, the total indirect cost of suicide was greater.^19^ Czernin et al. conducted a similar study in Switzerland, and analyzed the direct cost of attempted suicide in Basel.^20^ For 2003, the median cost per case was US$ 13,978. The parameters that were associated with high costs were age greater than 65 years, using a method with high lethality and great suicidal intent. Depression was associated with significantly higher costs than other diagnostic categories.^20^

While economic studies on suicide are generally based on national databases,^19^^,^^21^^,^^22^^,^^23^^,^^24^ studies on attempted suicide are based on hospital databases and generally only estimate the hospital costs. Estimates of the indirect costs are rarely available.^25^^,^^26^^,^^27^^,^^28^^,^^29^^,^^30^^,^^31^^,^^32^^,^^33^^,^^34^^,^^35^^,^^36^^,^^37^^,^^38^^,^^39^ Few of these studies include the costs of care outside hospitals or any information that allows characterization of the suicide attempt.^28^^,^^33^^,^^34^^,^^35^^,^^38^ The meager clinical descriptions and heterogeneity of the samples in these studies make comparisons of the findings difficult.^28^^,^^33^^,^^34^^,^^35^^,^^38^ From our review, there is no published estimate of the direct and indirect costs of attempted suicide in Brazil, in the scientific literature. On the other hand, we found assumptions regarding attempted suicide in a study on the cost of violence conducted by the Institute of Applied Economic Research (Instituto de Pesquisa Econômica Aplicada, IPEA). In this study, the average cost of hospitalizations registered as deliberate self-harm between 1998-2004 was estimated as R$ 507.00 (US$ 167.00), with an average hospital stay of four days. There was no description of which of these injuries were suicide attempts.^40^

When a study on attempted suicide is carried out, the clinical characteristics of these attempts need to be taken into consideration. The broad nature of the term “attempted suicide” can encompass distinct clinical situations within a single sample.^41^ In a study on a sample of patients admitted to a quaternary-level public university hospital because of suicide attempts, conducted by Rapelli in 2001, it was possible to distinguish three subgroups of individuals with distinct characteristics using the characteristic of suicidal intentionality: group A (impulsive-ambivalent), group B (marked intent) and group C (definite intent).^42^^,^^43^ Not only can clinical characteristics be assessed with regard to the possibility of being used as predictive factors for the outcome from attempted suicide, but also suicidal intentionality can be addressed as a significant risk factor for repetition of suicidal behavior and death due to suicide.^44^

## OBJECTIVE

In this light, the objective of the present investigation was to describe the direct and indirect costs relating to cases of severe suicide attempts treated at a general hospital in Brazil. To contextualize the magnitude of these costs, the costs of attempted suicide were compared with those of acute coronary syndrome (ACS), the disease with the greatest worldwide economic burden in terms of healthy years of life lost.^45^

## METHODS

### Design

This was a cost-of-illness study with a follow-up of three months.

### Subjects

The sample consisted of 17 patients who were consecutively hospitalized at a quaternary hospital responsible for medical care for highly complex clinical cases between June 2009 and December 2010, because of attempted suicide. To decrease the risk of grouping distinct recurrences in the same category, we chose to evaluate suicide attempts that are considered severe, with great suicidal intent^42^^,^^43^^,^^44^ and lethality.^43^^,^^46^ The inclusion criteria were as follows: the patients were at least 18 years old; had been hospitalized because of a suicide attempt; had a Beck Suicidal Intent Scale^48^ score greater than or equal to 18, which ranks as high suicidal intent, according to a locally performed study^42^ (see below); and had a score of 2 or higher on the lethality scale (see below). The exclusion criteria were a profile reflecting dementia; a diagnosis of delirium; psychotic mental disorders; or cognitive damage that impeded access to information.

The comparison group consisted of patients hospitalized during the same period at the same hospital with a diagnosis of acute coronary syndrome (ACS), defined as unstable coronary pathological conditions with a common etiology: thrombus formation, inflammatory processes and atheromatous plaques in coronary arteries. The pathological conditions included acute myocardial infarction and unstable angina.^48^ The comparison patients were aged 18 or older with a lethality scale score of at least 2. The same exclusion criteria that were applied to the attempted suicide group were also applied to the comparison group.

### Instruments

All of the subjects underwent a structured interview, with the aim of characterizing the sociodemographic and clinical profile. The following two instruments were also used:

Beck’s Suicide Intent Scale (SIS). This instrument enables a quantitative evaluation of the patient’s intention of dying. The items of the SIS include whether the patient stated that he/she hoped to die, whether the patient left a suicide note, the patient’s final acts in anticipation of death, and his/her reactions after having survived. The scale contains 15 items, and the final score ranges from 0 to 30 points.^47^ In the present study, we used a version of the scale that had previously been tested in Brazil.^49^ The median was used to define two groups: a group with lower suicide intent (score < 18) and a group with higher suicide intent (score greater than or equal to 18).^42^ We measured lethality on a Likert scale graded from 0 to 4 (0, no risk to life; 1, 25% risk of death without medical intervention; 2, 50% risk of death without medical intervention; 3, 75% risk of death without medical intervention; and 4, 100% risk of death without medical intervention) based on the clinical evaluation by the medical researcher. The ratings were based on an examination of the patient’s physical condition, the procedures used during the patient’s hospital stay (e.g. endotracheal intubation, nasal-gastric catheterization, stomach pumping, throat draining, artificial ventilation and tracheotomy, hemodynamic support and length of stay) and consultation with the attending physician. In the present study, scores of 2 or more points were considered to represent high lethality.

Mini-International Neuropsychiatric Interview (MINI). The MINI is a standardized, semi-structured diagnostic interview that leads to standardized psychiatric diagnoses based on the International Classification of Diseases, Tenth Revision (ICD-10) and the Diagnostic and Statistical Manual of Mental Disorders, Fourth Edition (DSM-IV).^50^^,^^51^

The direct costs were the summation of the hospital and out-of-hospital costs resulting from the event, determined via analysis of the medical records. These included the costs relating to the hospital stay, the health professionals mobilized for care, emergency room evaluation, medications used, diagnostic examinations, procedures performed (surgery, invasive procedures and other interventions), orthotics and prosthetics. The table of values from the Brazilian National Health System (Sistema Único de Saúde, SUS)^52^ was used as a reference for the amounts paid to the hospital for the procedures performed. The average cost of daily hospital care was calculated using the ratio between the total hospital costs and the length of hospital stay (in days). The indirect costs were based on the loss of the patient’s income and the income of the family members and caregivers for the duration of the illness. The values were estimated based on the patient’s salary per day (and that of the patient’s family) multiplied by the number of days absent from work.^9^^,^^53^^,^^54^ Early retirement and pension benefits were included in these costs when they were affected by the events studied.

### Procedures and data analysis

All of the patients admitted to this hospital due to suicide attempts were evaluated by the psychiatric liaison team. During the study period, this team interacted with the researcher via telephone to evaluate the inclusion/exclusion criteria and to collect data. Patients who met the inclusion/exclusion criteria were informed of the research and invited to voluntarily participate via a free and informed consent form. The subjects were registered for the study in the order of their arrival at the hospital. For each attempted-suicide patient who was included in the research, an ACS patient who was hospitalized during the same period was recruited for the comparison group. When two or more ACS patients were available, the case with the greatest sociodemographic similarity to the attempted suicide case was selected.

A follow-up evaluation was performed three months after the date of the acute event (suicide attempt or ACS) to estimate the indirect costs. The values were transformed into United States dollars (US$),^55^ on the admission date.

For analysis between the two groups, the Mann-Whitney statistical test was used to compare sociodemographic data, and the chi-square test was used to examine psychiatric and clinical comorbidities. The Mann-Whitney test and analysis of covariance (ANCOVA) with rank transformation adjusted for age were used for intergroup comparison of the direct, indirect and total costs.^56^

## RESULTS

During the study period, there were 587 psychiatric consultations for hospitalized patients. Of these, 26 were adults who had attempted suicide, and these patients were subjected to evaluation. Nineteen patients met the inclusion criteria of this study; however, two patients refused to participate. Thus, the sample of attempted suicide cases consisted of 17 subjects: 7 who had used a violent method (stab wounds, gunshot wounds or hanging) and 10 who had poisoned themselves. Table 1 shows the sociodemographic and clinical data on the attempted suicide group and the ACS group. There was similarity between the two groups in terms of demographic data, with the exception of the age range. The ACS group was on average 10 years older than the attempted suicide group (Table 1).

**Table 1.**
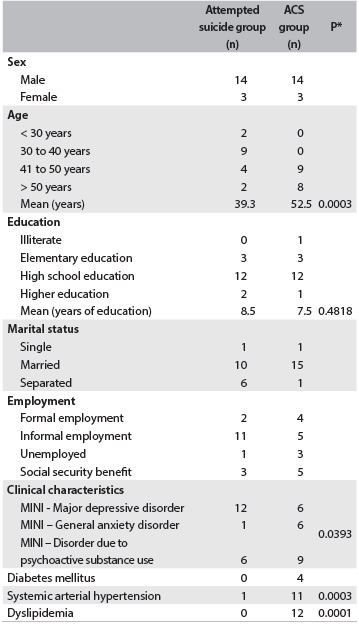
Sociodemographic and clinical data for the attempted suicide and acute coronary syndrome (ACS) groups

*Mann-Whitney test. MINI = Mini-International Neuropsychiatric

**Table 2.**
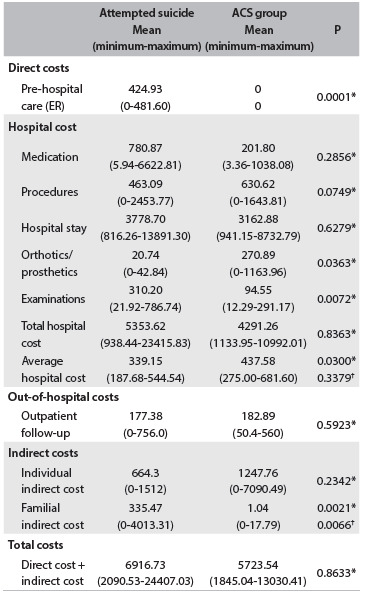
Direct and indirect costs (in US$) of the attempted suicide group and the acute coronary syndrome (ACS) comparison group

*Mann-Whitney test; ^†^Analysis of covariance (ANCOVA) test with rank transformation adjusted for age; ER = emergency room care.

The direct and indirect costs are presented in Table 2. The pre-hospital care cost included the costs of medical transfers carried out by the mobile emergency care service. None of the patients in the ACS group used this transfer service. In the attempted suicide group, 16 out of the 17 patients used such transfers, and these represented a significant difference in the pre-hospital care cost.

Although the total hospital cost did not differ between the groups, the ACS patients had shorter hospital stays (9.8 days versus 15.4 days for suicide attempts), thereby increasing the average hospital costs of the attempted suicide group (P = 0.0300). The ACS group had significantly higher hospital costs relating to orthotics and prosthetics. This difference resulted from use of stents (endoarterial prostheses), which cost more than R$ 2,000.00 (US$ 1,120.00) per unit, in treatments for patients with ACS. Meanwhile, rigorous laboratory tests and skull imaging examinations in cases of attempted suicide by exogenous intoxication (most of the subjects who resorted to exogenous intoxication were unconscious at the time when care was provided) contributed significantly towards the hospital costs of the attempted suicide group. The attempted suicide group had significantly higher indirect family costs than the ACS group.

## DISCUSSION

Our findings indicate that severe suicide attempts with high suicidal intent in which highly lethal methods were used presented total economic costs that were as high as those of ACS. Among the direct cost components, the hospital cost stood out, with daily hospital costs accounting for a large portion of the cost. Comparative analyses between the groups for the different types of costs (direct costs, indirect costs and indirect familial cost) indicated that there was greater indirect familial cost in the attempted suicide group.

The sample size of the study was limited by our decision to only include attempted suicides with clinical profiles of greater severity, which means that this study does not represent the range of suicide attempts seen at a general hospital. The small sample size limited intragroup analyses of factors such as the influence of mental disorders (or lack thereof) and any prior history of suicide attempts, on the final cost of suicide attempts.

Over the course of the study period, we noticed that the number of attempted suicide cases to be hospitalized decreased. This may have been due to rearrangement of the coverage areas of general hospitals in the city of Campinas, where this study was conducted, with the establishment of two new emergency care facilities (Ouro Verde General Hospital and Campo Grande Emergency Care). These two new hospitals cater for cases of lower complexity and they may be absorbing the demand from patients with lower degrees of clinical severity. We did not observe any decrease in the number of cases of greater clinical severity at our hospital.

According to the description in Rapelli’s study,^43^ over a 35-month period the hospital received 121 attempted suicide cases that needed hospitalization. Of these, at least 8 subsequently died, while 43 were of lower intentionality and clinical severity (scale of suicidal intentionality and clinical severity less than 18 points).

Over the 18-month period of our study, there were 61 assessments because of suicidal behavior. Comparing the numbers of hospitalizations due to suicidal behavior of high intentionality, there are significant differences between the 2005 study and ours: within the sample of patients hospitalized due to suicide attempts, Rapelli found that 16.5% of the patients had characteristics of high suicidal intentionality (n = 17).^43^ In our study, among the patients hospitalized because of suicidal behavior and who required psychiatrist consultation-liaison care, 27% of the cases showed characteristics of high suicidal intentionality (n = 19).

The fact that data gathering was performed at a university hospital also carries a bias. Hospitalizations at university hospitals typically generate higher costs resulting from the greater length of hospitalization and higher spending on materials and propaedeutic investigations. A study that compared the hospital costs relating to cases of intentional self-injury in different British hospitals showed that the costs were 33% higher when care was provided at university hospitals.^32^

The length of follow-up was also a limiting factor for the present study. The three-month period characterizes the acute phase for both of these pathological conditions. However, for a chronic and incapacitating disease like ACS, a three-month follow-up may underestimate the indirect costs.^57^ Therefore, the similarity between the two groups’ indirect costs found through the present study is restricted to a short period after the debilitating event occurred.

On the other hand, one strength of our study was its description of the indirect costs of attempted suicide. Few studies have examined this value. Here, the indirect costs of attempted suicide represented approximately 10% of the total cost over the three-month follow-up period. Longer follow-up periods may show a different relationship between indirect costs and total costs. In a study by O’Dea and Tucker, who used a follow-up period of 12 months, indirect costs accounted for 40% of the total cost of suicide attempts.^19^ Their finding highlights the importance of including an assessment of indirect costs in economic studies on suicidal behavior.

In our region, the medical pre-hospital service is carried out by SAMU (Serviço de Atendimento Médico de Urgência), a mobile emergency care service. The attempted suicide cases mostly needed some type of pre-hospital service because they involved unconscious patients, with evident perforating contusional injuries that presented the risk of hemodynamic instability. It is worth mentioning that in the present study, the cutoff for defining attempted suicide of high suicidal intentionality was that the patients did not ask for help. SAMU was called by the patient’s family and caregivers. On the other hand, in spite of the clinical seriousness of ACS, the prevalent symptoms (chest pain) warned the patients to look for medical care on their own or to ask their families for help. In these cases, the lesion was not intentionally caused by the patient, who had the desire to survive and to receive medical care as soon as possible. Thus, the cost of pre-hospital care in the attempted suicide group was significantly higher than in the ACS group. In other studies that we reviewed, there was no separation between the pre-hospital care and other costs.

In a study by Luna et al., the average cost per episode of suicidal behavior was US$ 12,831.00, with an average of 17.5 days of hospitalization. Within this value, the authors did not make any distinction between attempted suicide and death due to suicide. The inclusion of cases of death due to suicide and long stays in the intensive care unit may have been responsible for the large difference in hospital costs in comparison with our study.^25^ Meanwhile, in the study by D’Mellos et al., the cost of hospitalization in the intensive care unit for patients with intentional poisoning caused by antidepressants remained on average 2.5 times lower than that of our study.^28^

In reality, upon comparing the costs calculated in our study with those obtained from reviewing the literature, difficulties result from the different methodological options adopted. Some studies have included different age ranges in the same sample.^26^^,^^27^^,^^32^^,^^36^ For example, it is known that studies that analyzed the cost of suicidal behavior during adolescence have found relatively low costs.^37^^,^^39^ The majority of such studies only included a description of the hospital cost, and the elements considered in such evaluations were not homogenous among studies.

**Chart 1.**
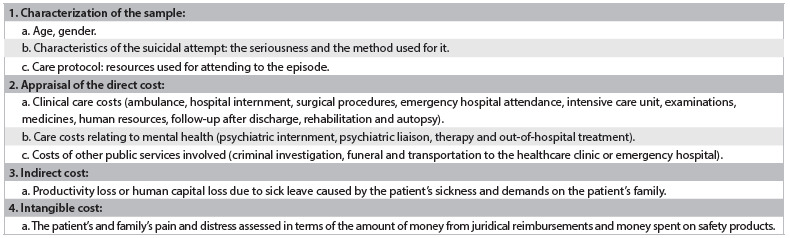
A suggestion for components to be included in a study on the costs of attempted suicide

Another methodological point worth highlighting is that few studies have included detailed clinical descriptions of suicide attempts.^25^^,^^28^^,^^30^^,^^34^^,^^35^^,^^37^ This omission risks including different autoregression occurrences in a single sample, with highly variable degrees of suicidal intent and lethality among the subjects. When less clinically severe suicide attempts are included, for example, shorter hospital stays are included, thereby resulting in lower hospital cost. This, together with the greater frequency of attempts with less clinical severity, lowers the average hospital costs. Chart 1 suggests some parameters that future studies should discriminate in order to allow adequate contextualization and comparison of the costs relating to suicide attempts.

One important finding from the present study is the difference in the indirect costs for families between the two groups. In this evaluation, we estimated the financial impact for caregivers who cease financial productivity in order to care for the family member who attempted suicide or who had ACS. The reason cited by family members of patients in the attempted suicide group for their absence from work was not the need to provide clinical care, but the need to give emotional support to the patient and their fear of a repeated suicide attempt. This did not occur in the ACS group.

Thus, over the short term (three months), family members of patients in the attempted suicide group suffered significantly greater socioeconomic impact than experienced by family members of patients with ACS. The considerable financial impact on the families of patients in the attempted suicide group reinforces the idea that suicidal behavior not only affects the individual, but also his or her social environment.

Bearing in mind the methodological restrictions of our study, we can claim that severe suicide attempts represent an economic cost similar to that of ACS, which is the disease with the greatest worldwide economic burden. This finding provides a sense of the economic impact of a suicide attempt and may help to make public health administrators aware of the need to prevent suicidal behavior. Simple economic strategies, such as the WHO SUPRE-MISS project can save many lives and much money in a given country.^58^ The secondary care for these patients is potentially more expensive and will not decrease the frequency of repeated suicide attempts or even of successful acts of suicide.

Moreover, economic studies in all formats (cost of illness, cost effectiveness, cost utility and other formats) help to measure the economic burden of diseases, and can estimate the resources used for treatment, or identify avoidable costs by means of prevention and promotion programs, with an influence on management initiatives in preventive programs. Despite the importance of cost-of-illness studies for healthcare planning, few data are available regarding the cost of suicide attempts. More studies with longer follow-up periods and larger samples are necessary in order to further substantiate these findings.

Although this study was restricted to an evaluation of economic costs, the human distress resulting from suicide and attempted suicide cannot be neglected. This distress, which is difficult to measure, should also be taken into account in public healthcare policy decision-making.

## CONCLUSION

In addition to the emotional impact, suicidal behavior has an important economic impact. Attempted suicide with a high degree of suicidal intent and using methods of high lethality has a total economic cost that is as high as that of ACS. The secondary care for these patients is potentially more expensive than prevention.
